# Comparison of Oncological Outcomes Between Radical Prostatectomy and Radiotherapy by Type of Radiotherapy in Elderly Prostate Cancer Patients

**DOI:** 10.3389/fonc.2021.708373

**Published:** 2021-07-19

**Authors:** Xiao-Xiao Guo, Hao-Ran Xia, Hui-Min Hou, Ming Liu, Jian-Ye Wang

**Affiliations:** Department of Urology, Beijing Hospital, National Center of Gerontology, Beijing, China

**Keywords:** localized prostate cancer, elderly men, radical prostatectomy, radiotherapy, survival outcomes

## Abstract

**Objective:**

We aimed compare the oncologic outcomes of radical prostatectomy (RP) with those of external beam radiotherapy (EBRT), brachytherapy (BT), or EBRT + BT (EBBT) in elderly patients with localised prostate cancer (PCa).

**Methods:**

Localised PCa patients aged ≥70 years who underwent RP, EBRT, BT, or EBBT between 2004 and 2016 were identified from the Surveillance, Epidemiology, and End Results database. Multivariable competing risks survival analyses were used to estimate prostate cancer-specific mortality (CSM) and other-cause mortality (OCM). Subgroup analyses according to risk categories were also conducted.

**Results:**

Overall, 14057, 37712, 8383, and 5244 patients aged ≥70 years and treated with RP, EBRT, BT, and EBBT, respectively, were identified. In low- to intermediate-risk patients, there was no significant difference in CSM risk between RP and the other three radiotherapy modalities (all P > 0.05). The corresponding 10-year CSM rates for these patients were 1.2%, 2.3%, 2.0%, and 1.8%, respectively. In high-risk patients, EBRT was associated with a higher CSM than RP (P = 0.003), whereas there was no significant difference between RP and BT or RP and EBBT (all P > 0.05). The 10-year CSM rates of high-risk patients in the RP, EBRT, BT, and EBBT groups were 7.5%, 10.2%, 8.3%, and 7.6%, respectively. Regarding OCM, the risk was generally lower in RP than in the other three radiotherapy modalities (all P < 0.001).

**Conclusions:**

Among men aged ≥70 years with localised PCa, EBRT, BT, and EBBT offer cancer-specific outcomes similar to those of RP for individuals with low- to intermediate-risk disease. In patients with high-risk disease, EBBT had outcomes equally favourable to those of RP, but RP is more beneficial than EBRT. More high-quality trials are warranted to confirm and expand the present findings.

## Introduction

Prostate cancer (PCa) is the second most commonly diagnosed cancer in men, with approximately 1.1 million cases worldwide; moreover, it accounts for 15% of all cancers diagnosed (1). A total of 60% of PCa patients are aged ≥65 years at diagnosis, and the number of patients aged ≥70 years at diagnosis is increasing. It is predicted that the number of elderly patients will increase from 585,000 to 778,000 between 2018 and 2030 (2). The latest consensus of the International Society of Geriatric Oncology on PCa management in elderly patients recommends that patients aged ≥70 years are managed according to their individual health status, and not according to their age. The “healthy” or “fit” older patients should have the same treatment options as the younger patients (3). Furthermore, several studies have reported that local therapy, such as radical prostatectomy (RP) or radiotherapy, can result in better survival outcomes than those of non-local therapy in elderly patients with localised PCa (4, 5). However, the survival benefits of these local therapies vary, especially for elderly patients (6–8). Furthermore, the comparative efficacy among these local therapies remains unclear. This study aimed to compare the survival outcomes of RP, external beam radiotherapy (EBRT), brachytherapy (BT), and EBRT + BT (EBBT) in localised PCa patients aged ≥70 years. Towards this goal, we identified patients from the Surveillance, Epidemiology, and End Results (SEER) database and conducted survival outcome comparisons.

## Patients and Methods

### Study Design and Population

**Figure 1:**
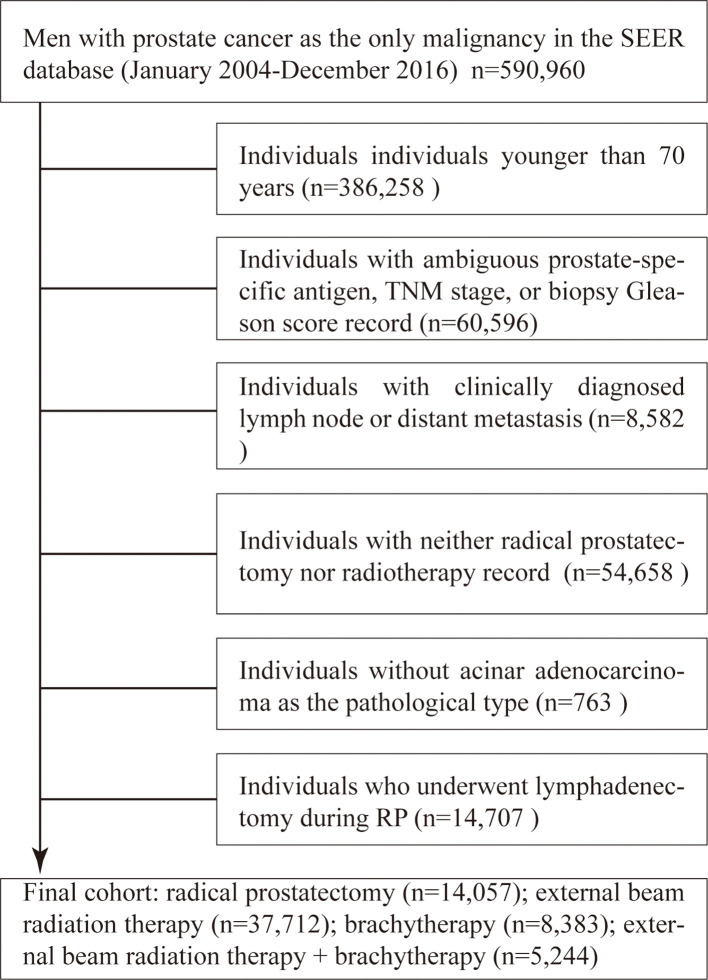
Patient selection flowchart.

Elderly men aged ≥70 years with localised PCa who underwent RP, EBRT, BT, or EBBT between 2004 and 2016 were identified from the 18th SEER tumour registries, which encompass approximately 26% of the US population. First, we screened 590,960 patients with PCa as the only malignancy. The exclusion criteria were as follows: (1) individuals younger than 70 years, (2) individuals with ambiguous prostate-specific antigen (PSA), TNM stage, or biopsy Gleason score (GS) record, (3) individuals with clinically diagnosed lymph node or distant metastasis, (4) individuals without RP nor radiotherapy record, and (5) individuals without acinar adenocarcinoma as the pathological type. RP patients with lymphadenectomy are pathological N0, and if they are compared against RT patients who are clinical N0, which is not equivalent. Therefore, individuals who underwent lymphadenectomy during RP were also excluded. The flow chart of patient selection is presented in **Figure 1**. Data on race, age, diagnostic year, preoperative PSA value, biopsy GS, clinical tumour stage (per the AJCC seventh edition), and follow-up periods were collected. The primary outcome measures were prostate cancer-specific mortality (CSM) and other-cause mortality (OCM).

### Statistics Analysis

Continuous variables are expressed as median (interquartile range [IQR]) and categorical variables as frequency and proportion. Between-group comparisons of clinicopathological features were conducted using the Kruskal–Wallis, Wilcoxon rank-sum, or Fisher’s exact test. Tumour stages were classified as ≤T2a, T2b, and ≥T2c. Biopsy GS was classified as ≤6, 7, and 8 to 10. Multivariable competing risks regression analyses were performed to evaluate the effects of different treatments on CSM and OCM (9, 10), with age, PSA, clinical T stage, biopsy GS, race, and diagnosis year used as covariates. The individuals who received both surgery and RT were place into the group that corresponds to the 1st treatment they received. Furthermore, the patients were stratified into subgroups according to the D’Amico risk classification (low- to intermediate-risk or high-risk). The cumulative incidence smoothed plots were generated for CSM and OCM according to the competing risks method, and the 10-year CSM and OCM rates were calculated. All statistical analyses were performed using R version 3.6.1 (The R Foundation for Statistical Computing, Vienna, Austria; www.r-project.org). All tests were two-sided, and P value of <0.05 was considered statistically significant.

## Results

Of the 65396 patients evaluated, 14057, 37712, 8383, and 5244 underwent RP, EBRT, BT, and EBBT, respectively. The RP group was significantly younger than the other groups (all P < 0.001). The BT group was more likely to have more favourable clinicopathological features, including lower PSA values (6.3 ng/mL), a higher percentage of biopsy GS of ≤7 (93.6%), and a higher proportion of ≤T2a disease (91.6%). Consequently, the BT group had the highest proportion of patients with low-to-intermediate-risk disease (86.7%). The median follow-up periods for the RP, EBRT, BT, and EBBT groups were 64, 64, 90, and 81 months, respectively. The baseline demographic and clinicopathologic characteristics of the four groups are summarised in **Table 1**.

**Table 1:** Baseline patient characteristics by treatment group.

	RP	EBRT	BT	EBBT	P Value
	(n = 14057)	(n = 37712)	(n = 8383)	(n = 5244)	RP vs. EBRT/BT/EBBT
Age (years)	72 [71, 74]	74 [72, 78]	73 [71, 76]	74 [71, 76]	<0.001/<0.001/<0.001
PSA (ng/mL)	6.7 [5.0, 9.5]	7.9 [5.6, 12.2]	6.3 [4.9, 8.6]	7.2 [5.2, 11.2]	<0.001/<0.001/<0.001
Race, n (%)					<0.001/<0.001/<0.001
Caucasian	11778 (83.8)	29573 (78.4)	6993 (83.4)	4084 (77.9)	
African	1026 (7.3)	4797 (12.7)	855 (10.2)	748 (14.3)	
Other	1143 (8.1)	2670 (7.1)	472 (5.6)	385 (7.3)	
Unknown	110 (0.8)	672 (1.8)	63 (0.8)	27 (0.5)	
Biopsy GS, n (%)					<0.001/<0.001/<0.001
≤ 6	11778 (83.8)	29573 (78.4)	6993 (83.4)	4084 (77.9)	
7	1026 (7.3)	4797 (12.7)	855 (10.2)	748 (14.3)	
≥ 8	1143 (8.1)	2670 (7.1)	472 (5.6)	385 (7.3)	
Clinical T stage, n (%)					<0.001/<0.001/<0.001
≤ T2a	10574 (75.2)	31133 (82.6)	7675 (91.6)	4095 (78.1)	
T2b	486 (3.5)	1825 (4.8)	246 (2.9)	399 (7.6)	
≥ T2c	2997 (21.3)	4754 (12.6)	462 (5.5)	750 (14.3)	
D’Amico classification, n (%)					<0.001/<0.001/<0.001
Low	2499 (17.8)	7286 (19.3)	4029 (48.1)	717 (13.7)	
Intermediate	6175 (43.9)	16240 (43.1)	3240 (38.6)	2423 (46.2)	
High	5383 (38.3)	14186 (37.6)	1114 (13.3)	2104 (40.1)	
Diagnosis year, n (%)					<0.001/<0.001/<0.001
2004 - 2007	3415 (24.3)	10895 (28.9)	3924 (46.8)	2102 (40.1)	
2008 -2011	4605 (32.8)	12516 (33.2)	2723 (32.5)	1763 (33.6)	
2012 - 2016	6037 (42.9)	14301 (37.9)	1736 (20.7)	1379 (26.3)	
Follow-up period (months)	64 [28, 99]	64 [30, 97]	90 [53, 119]	81 [45, 113]	

RP, radical prostatectomy; EBRT, external beam radiation therapy; EBBT, external beam radiation therapy + brachytherapy; PSA, prostate specific antigen; GS, Gleason score; IQR, interquartile range. Data are presented as n (%) or median [IQR].

**Figure 2:**
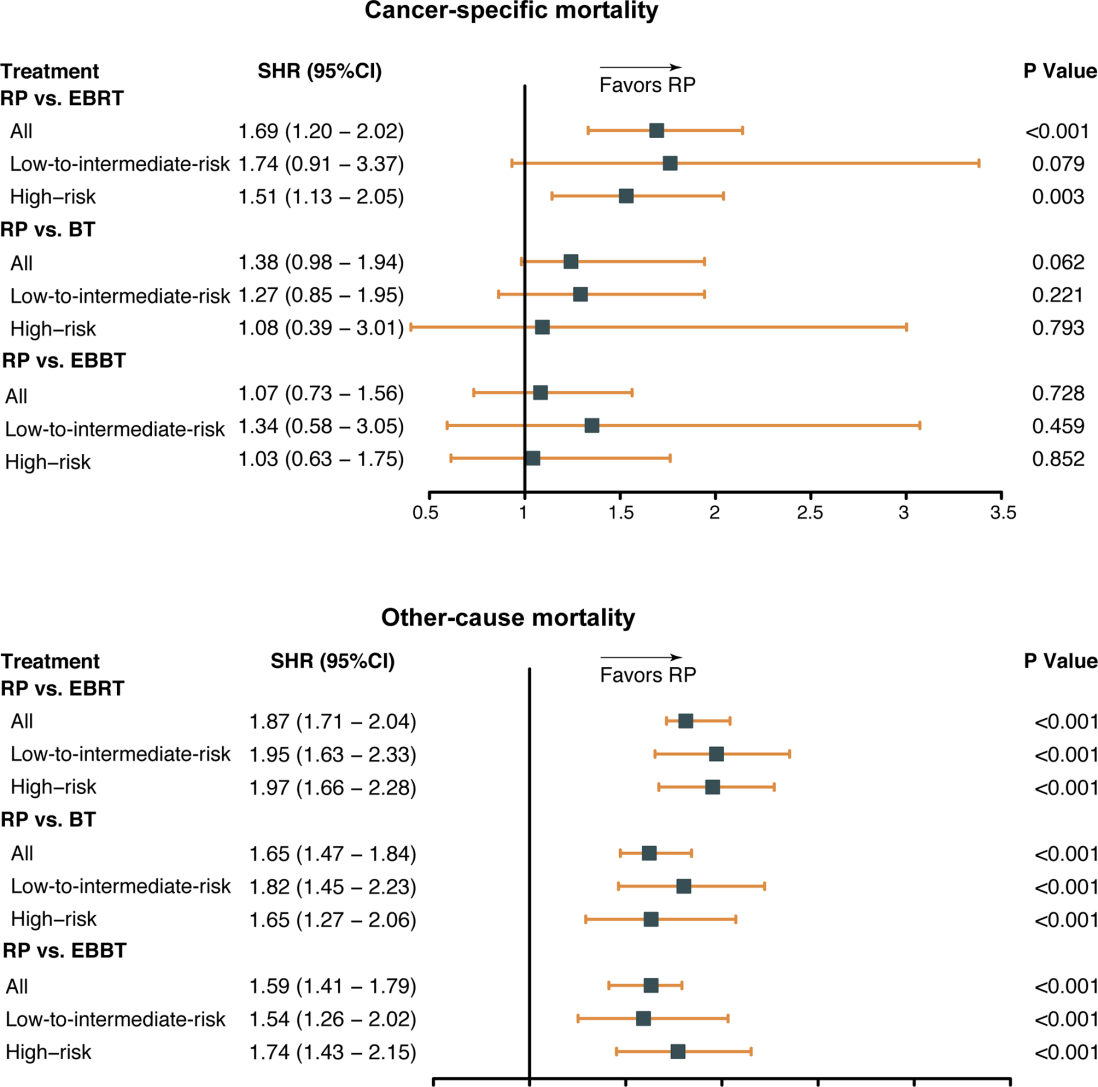
Subdistribution hazard ratio (SHR) for cancer-specific mortality by treatment modalities and risk categories. RP, radical prostatectomy; EBRT, external beam radiation therapy; BT, brachytherapy; EBBT, external beam radiation therapy + brachytherapy.

The results of multivariable competing risks analysis (**Table 2**) suggest that older age, earlier diagnosis year, ≥T2c stage, as well as higher PSA value and biopsy GS score were associated with higher CSM risk (all P < 0.05). In addition, EBRT was associated with a higher CSM risk (subdistribution hazard ratio [SHR] 1.69; 95% CI, 1.33–2.14; P < 0.001) than RP. However, the difference in CSM risk between RP and BT or RP and EBBT did not reach a significant level (SHR: 1.38; 95%; CI: 0.98–1.94; P = 0.062, SHR: 1.07; 95% CI: 0.73–1.56; P = 0.728). Subgroup analysis according to PCa risk categories (**Figure 2**) showed that RP had a lower CSM risk than EBRT for patients with high-risk disease (SHR 1.51; 95% CI 1.13–2.05; P = 0.003) but similar to those with low-to-intermediate-risk disease (SHR: 1.74; 95% CI: 0.91–3.37; P = 0.079). In addition, among all PCa risk categories, the CSM risk of RP was similar to that of BT or EBBT (all P > 0.05).

**Table 2:** Multivariable competing risk analysis for relative risk of death.

	CSM			OCM		
	SHR	95% CI	P Value	SHR	95% CI	P Value
Age	1.02	1.01-1.04	<0.001	1.10	1.09-1.11	<0.001
Diagnosis year						
2004-2007			Ref.			Ref.
2008-2011	0.90	0.80-1.00	0.057	0.89	0.85-0.93	<0.001
2012-2016	0.72	0.59-0.87	0.001	0.84	0.77-0.91	<0.001
Race						
Caucasian			Ref.			Ref.
African	1.17	1.01-1.36	0.040	1.25	1.18-1.33	<0.001
Other	0.54	0.42-0.68	<0.001	0.74	0.69-1.21	0.082
PSA	1.01	1.01-1.02	<0.001	1.01	1.00-1.01	<0.001
Biopsy GS						
≤ 6			Ref.			Ref.
7	2.06	1.75-2.43	<0.001	1.10	1.05-1.15	<0.001
8-10	6.33	5.37-7.46	<0.001	1.12	1.12-1.26	<0.001
Clinical T stage						
≤T2a			Ref.			Ref.
T2b	1.08	0.84-1.38	0.560	1.06	0.96-1.17	0.270
≥T2c	1.96	1.74-2.21	<0.001	1.09	1.03-1.16	<0.001
Treatments						
RP			Ref.			Ref.
EBRT	1.69	1.33-2.14	<0.001	1.81	1.71-2.04	<0.001
BT	1.24	0.98-1.94	0.065	1.62	1.47-1.84	<0.001
EBBT	1.08	0.73-1.56	0.728	1.63	1.41-1.79	<0.001

CSM, cancer-specific mortality; OSM, other-cause mortality; RP, radical prostatectomy; EBRT, external beam radiation therapy; BT, brachytherapy; EBBT, external beam radiation therapy + brachytherapy; PSA, prostate specific antigen; GS, Gleason score; SHR, subdistribution hazard ratio.

As for OCM, older age, earlier diagnosis year, African race, ≥T2c stage, higher PSA value and biopsy GS score, as well as three radiotherapy modalities, were associated with a higher OCM risk (all P < 0.001). Consistently, the subgroup analysis indicated that among all PCa risk categories, OCM was significantly lower in the RP group than in the other groups (all P < 0.001).

**Figure 3:**
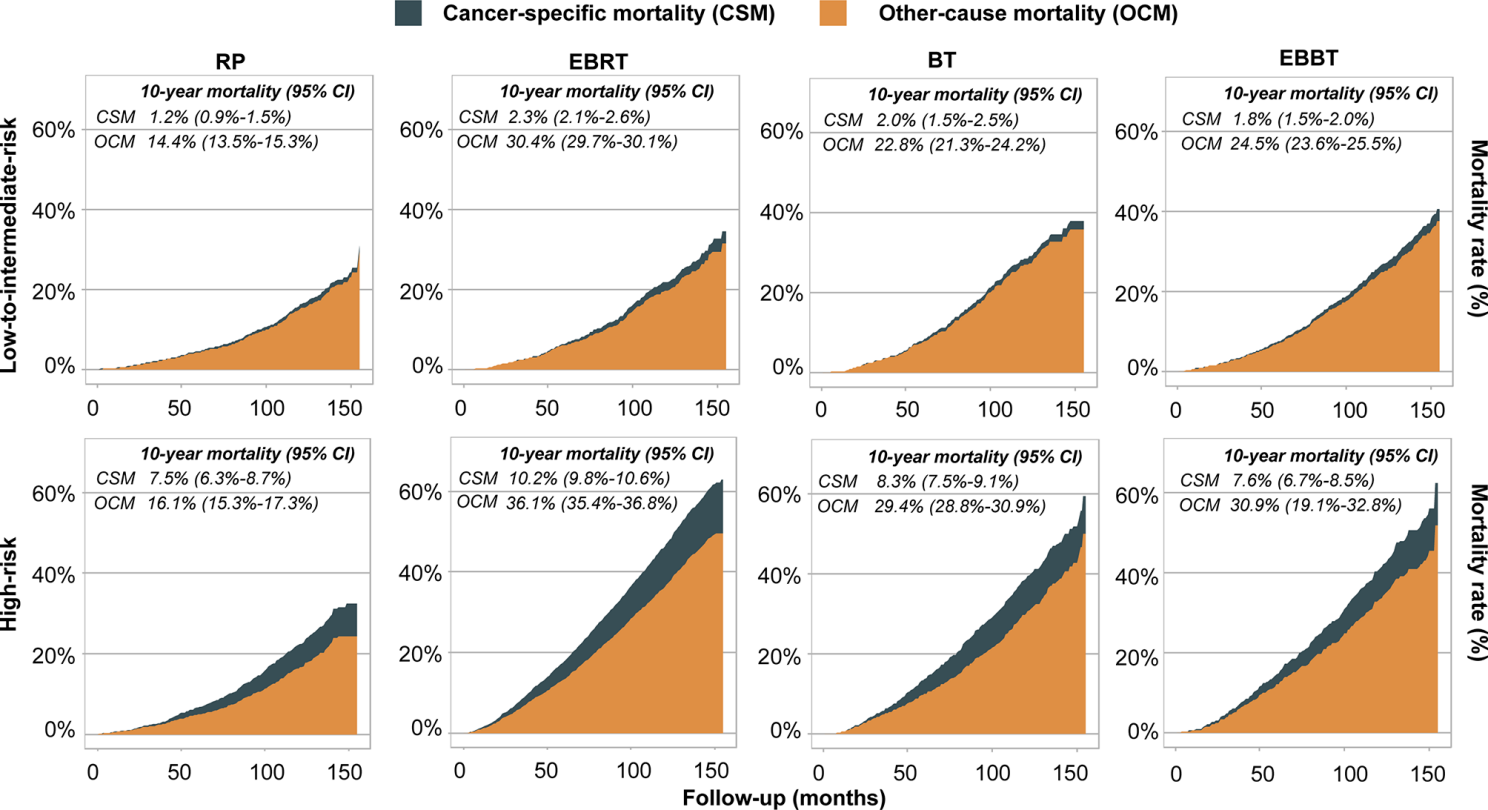
Competing risk models depicting cancer-specific and other-cause survival curves stratified by treatment modalities and risk classification. RP, radical prostatectomy; EBRT, external beam radiation therapy; BT, brachytherapy; EBBT, external beam radiation therapy + brachytherapy.

The CSM and OCM rates were calculated according to the treatment modalities and PCa risk categories (**Figure 3**). The 10-year CSM rates of patients with low- to intermediate-risk disease treated with RP, EBRT, BT, or EBBT were 1.2%, 2.3%, 2.0%, and 1.8%, respectively. The corresponding OCM rates were 14.4%, 30.4%, 22.8%, and 24.5%, respectively. For patients with high-risk disease treated with RP, EBRT, BT, or EBBT, the 10-year CSM rates were 7.5%, 10.2%, 8.3%, and 7.6%, respectively. The corresponding OCM rates were 16.1%, 36.1%, 29.4%, and 30.9%, respectively.

## Discussion

Over the decades, prostate cancer deaths in men 70 years and older are expected to almost double, whereas the overall mortality rate is anticipated to remain stable (2). The data highlight the importance of exploring the optimal management for PCa in elderly patients. However, the comparative efficacy among the local treatments for these specific patients remains unclear. In this study, the survival outcomes following RP, EBRT, BT, and EBBT were compared in elderly men with localised PCa.

We found that elderly patients who received EBRT had 1.69 times higher CSM risk than those who received RP. Further subgroup analysis suggested the association of RP with lower CSM risk in men with high-risk disease, but not in those with low-to-intermediate-risk disease. The results are in accordance with a study conducted by Wang et al. who evaluated the survival difference in the RP and EBRT groups of men aged ≥75 years with high-risk or very high-risk PCa based on the SEER database (11) and found that EBRT was associated with worse PCa-specific survival (HR = 0.533) and overall survival (HR = 0.453) than RP. However, another SEER study conducted by Abdollah et al. reported that for patients aged 70 to 79 years, radiotherapy was less effective than RP for those with low-to-intermediate-risk disease, but the effectiveness of two treatments was comparable for those with high-risk disease (5). It should be noted that most of their patients were diagnosed at the pre-PSA era, and the PSA value were therefore blank, which could potentially make substantial number of patients who actually had high-risk disease to be classified into the low-to-intermediate-risk type, and thus deviated the results. As for the comparative effectiveness of RP versus EBRT in low- to intermediate-risk PCa, the 10-year follow-up of the ProtecT trial, of which 60% and 40% of the population had low- and intermediate-risk diseases, respectively, found no difference in survival outcomes between the RP and EBRT (12). However, the trial only includes patients aged ≤65 years.

BT is another type of radiotherapy. The current study found that for PCa patients aged >70 years, there was no significant difference in CSM risk between those treated with RP and BT. Subgroup analysis by risk category found consistent findings. Arvold et al. used the data of 8839 patients to estimate the cancer-specific mortality following RP or brachytherapy in men with low- or intermediate-risk PCa. They found no significant difference in the risk of cancer-specific mortality between two treatments after a median follow-up of more than 4 years (13). Additionally, Zhou et al. assessed the biochemical relapse-free survival time (bRFS) and clinical relapse-free survival (cRFS) time after RP or BT for patients with T1c-T3a localised PCa. The results indicated that BT produced equivalent bRFS and cRFS rates compared with RP within 5 years of follow-up (14). However, the CSS and OS were not reported in their study. Although these studies were not tailored for older patients, the age range in both RP and BT arms extended to above 80 years, suggesting some relevance to this population.

A few recent studies reported that EBRT combined with BT had comparable and even superior therapeutic effectiveness to those of RP. For example, in a NCDB study, Ennis et al. compared EBBT to RP with or without adjuvant radiation therapy in men with high-risk PCa and found no significant difference in OS between the two arms (15). Muralidhar et al. compared the data following EBBT or RP with or without ART based on the NCDB and SEER database and reported that the two kinds of therapy provide equivalent OS and CSS for men with GS 9-10 PCa (16). In addition, Kishan and colleagues retrospectively analysed the data of 1809 men with GS 9-10 PCa and found that those who underwent EBBT were at significantly lower risk for 5-year CSS and distant metastasis than were those who underwent RP (3% vs 12% and 8% vs 24%, respectively, all p<0.001) (17). However, none of them were tailored for elderly patients. Our results suggested that RP and EBBT are associated with similar a CSM. The 10-year CSM rates of RP vs. EBBT were 1.2% vs 1.8% in the low-to-intermediate-risk subset, and 7.5% vs 7.6% in the high-risk subset, respectively. These findings confirmed the efficacy of EBBT in patients 70 years and older.

The present data provided information that may guide the management of elderly men with PCa. EBRT, BT, and EBBT offer comparable cancer-specific survival outcomes to RP in patients with low-to-intermediate-risk disease. In high-risk patients, RP has a better performance than EBRT, but not than EBBT. However, the percentage of men with localised PCa undergoing surgery rather than radiation has dramatically increased in recent years (18). This reinforces the need for patients to seek opinions from both a urologic oncologic surgeon with expertise in RP and a radiation oncologist with expertise in brachytherapy. Notably, despite our results suggesting that BT had similar performance to RP in patients with high-risk disease, BT as monotherapy is currently not the standard management for men with high-risk PCa. Indeed, high-risk disease only accounted for the minority of the BT group (13.3%). A small sample size of high-risk patients in the BT group coupled with other unknown confounding factors, such as androgen suppression therapy (ADT), reminds us that this part of the result should be treated with caution. In the present study, OCM was generally higher in radiotherapy modalities than in RP. One possible reason is that the “vulnerable” or “frail” patients with major complications were more likely to receive radiotherapy. Furthermore, ADT is more likely to be combined with radiotherapy (19). Androgen suppression is associated with some complications, such as metabolic syndrome, cardiovascular morbidity, mental health problems, and bone resorption, that could potentially increase OCM risk (20, 21).

Even though our study analysed a large sample of real-world data, the results should be interpreted in light of its limitations. First, as in all findings originated from retrospective data, there was a lack of randomisation between groups according to the baseline characteristics. Second, our study did not include information on hormonal therapy. Moreover, the doses delivered in radiotherapies were not reported owing to the nature of SEER. These could change the treatment response and potential complications. Third, patients in the RP group may have been “healthier” than those in the other groups. Although CSM was adjusted for OCM, comorbidities that potentially affected CSM cannot be ruled out, and they might have influenced the final results. The impact of treatments on OCM cannot be determined in the present study because we could not assess the frailty and comorbidity of patients due to the nature of SEER. Forth, the present study is brief, given that the cancer-specific outcomes besides CSM, such as biochemical free survival, disease-free survival, and distant metastases, which reflect the response of the tumour to treatments, were not reported by SEER. Hence, we were unable to perform analyses regarding these outcomes. Fifth, the patients with lymphadenectomy were excluded from the present study, which could potentially weaken the comparative effectiveness of RP. Because the patients may receive an expanded RT, which include lymph node field.

In conclusion, among localised PCa patients aged ≥70 years, EBRT, BT, and EBBT offer similar cancer-specific outcomes compared with RP for individuals with low- to intermediate-risk disease. In patients with high-risk disease, EBBT had equally favourable outcomes to RP, but RP is more beneficial than EBRT. It is worth noting that the limitations of the present study indicate that our findings should be interpreted with caution. More high-quality trials are warranted to confirm and expand our findings.

## Data Availability Statement

Publicly available datasets were analysed in this study. This data can be found here: https://seer.cancer.gov/.

## Author Contributions

Conceptualisation, X-XG. Methodology, H-RX. Software, X-XG. Validation, J-YW. and H-MH. Formal analysis, X-XG. Writing—original draft preparation, X-XG. Writing—review and editing, ML. Supervision, ML. All authors contributed to the article and approved the submitted version.

## Funding

This study was supported by the Beijing Hospital Clinical Research 121 Project (BJ-2020-171).

## Conflict of Interest

The authors declare that the research was conducted in the absence of any commercial or financial relationships that could be construed as a potential conflict of interest.
